# The Prevalence of Violence Against Iranian Women and Its Related Factors

**DOI:** 10.5539/gjhs.v7n3p37

**Published:** 2014-10-28

**Authors:** Alireza Nikbakht Nasrabadi, Nahid Hossein Abbasi, Neda Mehrdad

**Affiliations:** 1 School of Nursing and Midwifery, Tehran University of Medical Sciences, International Campus (TUMS-IC), Tehran, Iran; 2 Islamic Azad University of Ahvaz, Ahvaz, Iran; 3 Endocrinology and Metabolism Research Center, Endocrinology and Metabolism Clinical Sciences Institute, Tehran University of Medical Sciences, Tehran, Iran

**Keywords:** Iran, prevalence, violence, women

## Abstract

**Background:**

Domestic violence against women is a public health problem with negative consequences, and it is an intractable and widespread problem. This type of violence affects the stability of the family.

**Aim:**

The aim of this study was to estimate the prevalence of any violence against women referring to health centers and explore the associated risk factors with violence in Ahvaz, Iran.

**Methods:**

A cross-sectional study was conducted on randomly chosen samples of 368 married women aged between 15-55 years in 2013. The samples were divided to two groups, with abused experience and without abused experience. The data were amassed by questionnaire form.

**Results:**

The prevalence of violence against women was found to be around 63.8%, among them 58.8% were emotional abuse. The majority of women (84%) had never gone to a counseling center. Findings show 47% of women were silent, 27% got in a fight, 7% screamed, 6% abused their children, and 5% threw things when occurred violence against them. Experience of violence in women correlated with the marriage age of woman, numbers of children, and difference of marriage age between couple, marriage age of men, employed women, uneducated women and the rate of drugs use in their husbands.

**Conclusions:**

Nurses and other health care providers can and should play a major role in empowering women living with violence and promote education, social policies and attitudes that proactively prevent violence.

## 1. Background

Violence against women is a universal reality and is common in all countries, cultures and societies. It is at not rate acceptable or justifiable and is to be considered as one of the major women health problems. It is not only a women’s dominant problem but also a major issue in women’s human rights (WHO, 2013). The term violence against women includes forms of violence such as sexual partner violence and non-sexual partner violence. Evidence shows both a form of violence is widespread throughout the world, and it affects women’s physical, sexual and mental health. These findings send the world a very vigorous message that violence against women is not a trivial subject related to some societies; rather it is a universal problem and challenge whose prevention seems to be vital (WHO, 2011; WHO, 2013). Women are significantly more likely than men to be perpetrators of dating violence during young adulthood (38% vs. 19%) (Jain et al., 2010). Based on the World Health Organization’s report, the rate of violence against women is as following: Eastern Mediterranean countries (37%), African countries (36.3%) American countries (29.8%), European countries (25.4%), Western Pacific (24.6%) and other countries with high salaries (23.2%) (WHO, 2013).

Violence against women is a multi-dimensional phenomenon which is defined based on ethical, cultural and legal attractions and is an activity or attitude which hurts women. In fact, it is a real physical, sexual, emotional, social and economic threat and is known as a global health challenge. Such a behavior can secretly or openly take place through threat, compulsion or absolute free will and freedom privation (WHO, 2011; Nashwan, 2014). The side effects of violence against women in family do not just swipe the women, but they impose serious hurt to all family system and all bulk of the society (Razaghi et al., 2013). In other hand Violence in family beats destructive consequences. The evidence shows that %30 of women in the U.S are treated violently by their husbands, and less than half of them reported to the police (Flauger, 2014). The boys witnessing their mothers being misbehaved will misbehave their wives. In addition, the girls raised in such families can better tolerate their husbands’ violence (Rada, 2014). Nearly all of the victims of violence reported that the most recent experience of abuse influenced their current well-being and life management (Poutianen & Holma, 2013). Base on a study most of the violence victims were women whose age ranged from 21 to 40, who had young children and spent almost five years tolerating violence without any effective support. The findings show that the risk of behavioral problems and emotional damages are increasing in those children who witnessed violence. Moreover, when they are adult, the emergence of mental disorders is more probable (Co-ordinated Action Against Domestic Abuse, CAADA, 2012).

In healthy people program, different ways are proposed to organize, screen and prevent this problem. Studies conducted in Iran also confirm this phenomenon and its consequences (Razaghi et al., 2013).

The expenses spent to remedy violence side effects (physical and so on) are really considerable. Based on the statics of world health organization, this annual amount is over 5.8 billion dollars (Flaugher, 2014). Interventions for violence against women include both interventions focused on women such as strategies like education, support and psychological counseling and treatment for women at risk and interventions focused on health care providers such as nurses in order to train and enable them in diagnosing and managing violence against women (Soleiman Ekhtiari & Ahmadi, 2011).

Since most of the studies conducted on violence against women in Iran are limited to counseling centers and Forensics, and became one of the attraction of mother and child health care in health centers is to review violence against women and spouse abuse, the present study was, therefor, allocated to women who came to receive health services in health centers.

Considering statistic from Iranian researchers and comparing it with other findings from researchers from other parts of the world, universality of the issue, the importance of handing violence down from one generation to another, its effect on the life quality of women as a cornerstone of the family, and variety of tribes and clans in Ahvaz, the present study was conducted to determine the prevalence of violence against married women visiting health centers and to determine its related factors. The result from this study can be applied in different perspectives such as planning to promote the family health, protecting women as a vulnerable community and in social and cultural scopes by the nurses.

## 2. Methods

### 2.1 Study Population and Sampling

The present study is a cross-sectional, descriptive-analyzing, one which started in February 2012 and ended in May 2012. The purpose of this study is to determine the prevalence of violence against in married women visiting Ahvaz’s health centers. After the pilot study, sample was obtained by frequency (p=alpha =0.5, 65.7%). 368 married women whose age ranged from 15 to 50 were chosen. The criteria to participate in the study was to be married and the couples are married for more than one year.

### 2.2 Research Tools

The data gathering tool in this study was a questionnaire form. The questionnaire included demographic factors of women (age, educational level, occupation, marriage age, the length of marital life, and the number of the children) and husband’s information (besides all above mentioned factors, satisfaction of child’s gender, smoking cigarette or using alcohol, the amount of salary and drug addiction were included).

To determine reliability, content reliability and simultaneous test was used. Using the test person’s narrative correlation coefficient (78%) was obtained. Because in Ahwaz there are two health centers-East and West- each of which covers various health centers, multi-stage sampling (cluster-randomized) was used. The samples were categorized in two groups: with or without experiencing abuse.

### 2.3 Data Analysis

The data were analyzed using SPSS 16 software and descriptive statistics (percentage, frequency to determine the rate of physical, mental, sexual and economic abuse) and inferential statistic (t-test to compare the mean of variables between two groups of with or without experiencing abuse and Chi-square, to determine the significance of nominal variables) with the confidence of 96%.

### 2.4 Ethical Considerations

After choosing the samples based on the participation criteria, the researcher visited the participants, explained the purpose of the study, confirmed the confidentiality of the gathered data, and registered their consent in special forms. Informed consent of all participants was obtained and a regulation of the Declaration of Helsinki was followed throughout the study.

## 3. Results

### 3.1 Demographic Status of Participants

In this study, 368 married women were interviewed. The age average was 36.8±12.3 and most frequency was for ages ranging from 36-40 in all samples. The age average for the group experiencing violence was 28.96±5.3, and for the group not experiencing violence was 29.49±4.2. The age average of the husbands for the group experiencing violence was 38.3±7.1, and for the group not experiencing violence was 31.5±5.5. Of all the participants, 12.4% were university educates, 49.6% had high school diploma, and 38% of them were lower than diploma. 10.8% of them were employed, and 89% of them were householders. The average of marriage age was 21.2±5.4, and 6.98% had divorce experience. 10.8% of them married at the age of 15 to 19, 36.9% at the age of 20 to 24, and 52.3% married when they were over 25 years old. The average age of the mutual life was 20±12.3. The average number of the children was 9.2±2. 9.8% of them had no children. 23.4% had two children, 29.6% had three children, and 37.2% had more than four children. 16.8% had daughters, 12.9% had sons, and 70.3% had daughters sons.

### 3.2 Frequency of Different Types of Violence in Subjects

The result showed that 63.2% of the women participating in this study experienced violence. Violence includes physical violence 34.4%, mental violence 58.8%, sexual violence (from their husbands) 34.2%, and life- threatening violence 12.2% (Table 1). The most frequent type of violence is mental violence. Some types of physical violence include pushing, hitting (slapping, punching and kicking), threatening with a firearm or cold, beating (with sticks, belts,) and burning, breaking orangs. Mental abuse includes terror, threatening to divorce, deprivation of parental and relative visit and telephone and verbal communication. Sexual abuse includes indifference and apathy, forced sex, unconventional sex and forced unlike sex (Table 2). The most frequent forms of physical abuse such as pushing and beating was respectively 65.5% and 76.7%, mental abuse such as deprivation of visiting friends and suspicion was 65.8% and 74.5%, economic abuse such as the cost of austerity is 47.7%, and sexual abuse such as indifference was 35.1%. Due to the fact that most of the women lived in the nuclear families, but they believed that it was the man in the family (78%) who had the power.

**Table 1:** Absoluteb and relative frequency of different types of violence in subjects

violence	Frequency	Number	Percentage1
Physical	experiencing	165	43.4
	Not experiencing	203	55.1
Mental	experiencing	217	55.1
	Not experiencing	151	58.8
Economic	experiencing	126	41.0
	Not experiencing	242	34.2
Sexual	experiencing	44	12.2
	Not experiencing	324	88.0

**Table 2:** Frequency distribution of different kinds of violence in married women experiencing violence from their husbands

violence	Frequency
Physical: pushing	65.5
Beating	65.7
Breaking organs	23.3
Threating with cold arms	37.9
Threating with firearms	-
Burning	21.8
Mental: terror	11.8
Suspicion	74.5
Threating to divorced	13.9
Depuration of family relation	42.9
Depuration of friends relation	65.8
Economic: barring from employment	16.7
Lake of financing	17.7
Hard decision on spending	47.7
The income of the spouse	19.6
Sexual: indifference	35.1
Forest sex	9.1
Unconventional sex	0
Obligation in unlawful sexual relation	0

**Table 3:** Distribution of mean standard deviation and the significance level of variables associated with Variable

Variable	Experiencing abuse	Mean	Standard deviation(SD)	p

	Positive	18.6	2.23	P<0.001
Marriage age	Negative	22.2	2.54	
	positive	2.3	1.52	P=0.03
Number of children	Negative	1.2	1.14	
	Positive	6.8	7.43	P=0.3
The length of marital life	Negative	8.1	7.31	
	Positive	6.2	3.64	P<0.003
Age difference with husband	Negative	4.3	3.35	
	Positive	28.3	5.40	P=0.001
Women’s marriage age	Negative	25.2	5.91	
	Positive	93.5	3.7	P=0.004
Husband’s marriage age	Negative	27.9	5.9	
	Positive	89.6	2.32	P=0.001
The amount of monthly salary	Negative	305.35	4.7	

### 3.3 Adverse Actions Against Women in Association With Demographic Status

The frequency of adverse actions against women in terms of demographic status of women and their spouses were examined and analyzed using t-test. The obtained results confirmed there is a significant relationship between women’s marriage age variables (p<0.001), the number of children (p=0.03), age difference between spouses with over 10 years (p<0.003) spouse’s marriage age (less than 20 and more than 40) (p=0.001) and the experience of women’s adverse actions.

To review the relation between nominal variables and adverse action, chi-square test was used. The results showed that there is a significant relation between the variables of women’s employment (p<0.001), women’s educational level (p=0.004) (most of the women having educations higher than diploma were located in the group not experiencing violence), husband’s edition to drug and alcohol (p<0.003) and women’s experience of adverse action. There was also no significant relation between the variables of husband’s satisfaction with the child’s gender (p<0.2), husband’s smoking (p=0.3, p=0.6) using chi-square test and the experience of violence by women. The results also showed that most women’s reaction (47%) against husband’s silence and debate was (27%) screaming was (7%) and abuse of children was (6%). After adverse action, most of them refuge to their mothers (%30), other members of the family, fathers, friends, children, and other relatives.

## 4. Discussion

The results of the reviews about violence against women show that in order to express this problem, various terms such as violence, espouse abuse and misbehavior are used. All of these terms represent unconventional behavior towards women (Rodringuez et al., 2012; WHO, 2013; Nashwan, 2014). The results of the conducted studies show that there is widespread violence against women in different countries. In Iran, many studies have been conducted about this problem. The results show that the prevalence of violence against women varies between 27% to 83% (Khani et al., 2011; Sadegi Fasaei, 2011; Aghakhani et al., 2012; Jamshidimanesh et al., 2012; Mohammadi & Mirzaei, 2012).

In this study, the relative frequency of violence prevalence in women visiting Ahwaz’s health centers, based on obtained findings, was 63.2%. Aghakhani et al. (2012) also stated that 89.3% of women coming to forensics in Uremia experienced physical violence from their husbands. A study in Ethiopia indicated, the prevalence of domestic violence was 78.0%. About 73.3%, 58.4% and 49.1% of women reported different forms of psychological, physical and sexual violence, respectively (Semaheg et al., 2013). There is a relationship between mental health and violence against women; in such a way that the rate of depression, anxiety, dray misuse and suicidal thought are higher in such women (Trevillion et al., 2012). In Pakistan, Karmaliani et al. (2009) showed that 51% of women confronted with violence suffer from physical problems such as nervous headache, stomachache, scald and venereal illnesses Requesting psychological medication, fatigue, beating and ill-speaking, sleep disorder and anxiety. Depression was the most prevalent one (Watts & Mayhew, 2004; Joyner & Mash, 2012). In Iran the women deciding to divorce received physical misbehavior (62.5%) and sexual misbehavior (48.5%) from their husbands (Foroutan & Jaddid Milani, 2008). Moasheri et al. (2012) revealed the prevalence of violence against women to be 42.3%. Watts and Mayhew (2004) showed 13% to 49% of married women in Africa suffer from violence. Also conducted study in Turkey which claimed 44.4% of the women under the studies suffered from violence from their husbands in a period of their lives (Bener et al., 2010). The results of a study conducted by Mohammadi and Mirzaee (2012) showed that the highest rate of violence against women was mental violence and, the lowest violence was economic violence. There is also a significant relationship between men’s authoritative attitudes about their functions and violence against women. Studies also reported the prevalence of sexual violence (19% to 45%) among women at the age of 16 to 45 in some of the provinces in India (Eby et al., 2008; Babu & Kar, 2009).

As one can see, the prevalence of violence is higher in some societies and lower in the others. This difference could be due to the number of the samples or cultural difference in different societies (Hesegawa et al., 2005; Breiding et al., 2008; Eby et al., 1995; Kharboush et al., 2010; Semaheg et al., 2013). Many studies are conducted to survey different aspects of violence, some of which like that of Watts and Mayhew (2004) showing prevalence of physical and mental violence among women admitted to the emergency department (70% to 75%) are consistent with the result of the present study. The statistical difference could be due to the so social difference or the place of the both studies.

Concerning the second hypothesis of the study, the findings clarified that followings are effective in women’s experience of violence in the study: employment, economic conditions, level of education, marriage age, satisfaction of the child’s gender, age difference of age at marriage, and the number of the children. The obtained results showed that there is a significant statistical relation between variables such as employment, economic condition, the level of education, marriage age and occurrence of violence against women.

The above-mentioned studies are also consistent with the result of the present study. Bostak et al. (2009) declared in a qualitative study the economic condition and young women are more exposed to violence. The result of a study by Babapour et al. (2007) showed that pregnant mothers in Tabriz experience physical violence (23.6%). There was also a significant relationship between husband’s educations, health insurance type, husband’s attitude toward unplanned pregnancy, insufficient revenue in the eyes of the mode and husband’s using alcohol. Rodringez et al. (2012) also studied the rate of violence against female nurses, and the results showed that the rate of violence was 33%, of which 75.1%, related to mental violence was. There was, moreover, a significant relation between nurses financial independence in occurrence of violence. Khani et al. (2010) believed those husbands who have less access to power sources such as income, education, and employment or take the lower positions than their wives use violence as an instrument to gain power and position. Based on the results of a study by Aghakhani and et al., There was a significant relationship between physical violence and husband’s employment; that is, those husbands who did not have a permanent job showed more physical violence (2012). Kamat et al. (2010) argued the rate of husband’s physical violence against women in Goa is reported as 26.6% which has a meaningful relation with women’s level of education, lower age of marriage, and their husband, using alcohol. In this study Unemployment, having a populated family and marriage age are among predisposing factors of violence. Based on the results of a study conducted by Balali Meybodi and Hassani (2009), most of the violence from the viewpoint of the violence-affected women was inappropriate economic condition and their husbands’ job.

In the present study, it is evident that there is no significant statistical relationship between smoking cigarette, drinking alcohol and men’s violence towards their wives. But husbands’ addiction to narcotics is of those variables affecting women’s violence.

The research studies conducted concerning this issue in Iran and other countries are consistent with the findings of the present study. In fact, because of the destructive effect of addiction on behavioral, economic, mental and temperamental aspects, these findings were not unexpected. Balali Meybodi and Hassani (2009) considered addiction as one of the effective factors on violence occurrence. Bener et al. (2010) conducted a study in Turkey showed the relation between using narcotics alcohol and physical violence. The result of the present study also showed that based on the most of the participants (87%) none of the health care provider asked about spouse violence. This subject is considerably important. In this regard, studies in other part of the world showed that most of the nurses working in health centers are inadequately equipped to discover and interfere with violence against women (Sundborg et al., 2012). In this scope nurses can offer their supports which include identifying violence types, assessing and reviewing, reporting violence, programming and referring women to counseling centers. Nurses play a vital role in enabling those women who suffer from violence and promoting education, policy, attitude and preventing violence (Blideman, 2010; Ker & Mcconvill, 2012; Usta et al., 2012;. Rodringuez et al., 2012).

In this regard, the results also showed that most of the women experiencing violence never came to counseling centers (84%). In a study conducted in Egypt, it showed half of the Egyptian women suffered from physical violence (beating) by their husbands, and most of them kept silence and requested no help in such circumstances (Kharboush et al., 2010). One of the major concerns of the woman treated violently not to mention the violence is the fear to lose their children, the findings of the study reveal although the mother keeps on living in such cases, the frequently negative consequences on the child witnessing the violence are highly taken into consideration (CAADA, 2012).

Therefore, it can be inferred from the findings of the present study and other studies conducted to unveil the same issue in other parts of the world that, despite cultural, economic and technological differences, regardless of prevalence statistical differences or its related factors in human societies, the prevalence of violence against women is undeniable. A society who pays close attention to its health and the function of future generation must stand against those factors which pave the way for violence and abuse occurrence among the family members and sustain the health of the future-making generations to attain humane and divine ideals and be ready to elevate the nation.

## 5. Conclusion

In general, it can be said that widespread violence against women is a global challenge whose prevention seems to be vital. The best time of prevention refers to the school time when children are trained to respect each other’s rights. In other words, passing comprehensive laws can prove helpful in decreasing the consequences of violence occurrences through standing against domestic violence, betterment of socio-economic condition and preaching public education through media, especially the internet and the presence of medical-psychological centers. It is also recommended to stand fundamentally against such a social crisis through offering appropriately training teenagers and youngsters in educational centers. This can be effective in decreasing socio-psychological effects and the imposed expenses resulted from physical-mental consequences. The necessary matter which can help this ideal come true is to enable nurses to play a wider role in societies, which is only feasible through nurses’ presence in health centers. The nurses must have a true understanding about the complicated phenomenon of violence against women in order for nurses to be able to protect women and to play the precious role of giving health services in all fields of preventing violence. Training the needed skills for prevention and stopping violence is vital for both women and nurses. In other words, the need to increase knowledge concerning violence seem to be necessary to lessen physical, sexual, mental, emotional and economic injuries associated with this issue.
